# Attention-Deficit/Hyperactivity Disorder (ADHD) and High Risk Behaviors

**DOI:** 10.5812/ijhrba.12817

**Published:** 2013-06-26

**Authors:** Nour-Mohammad Bakhshani

**Affiliations:** 1 Department of Clinical Psychology, Children and Adolescent Health Research Center, Zahedan University of Medical Sciences, Zahedan, IR Iran

**Keywords:** Attention Deficit Disorder with Hyperactivity, Risk Behavior

High risk behaviors (HRBs) can impact public health. HRB refers to any kind of behavior or reaction that can potentially harm psychological and biological aspects of an individual. These behaviors are hazardous to several areas of human development which include: health, performing duties or tasks commensurate with developmental stage, playing expected social roles, learning the skills, feeling competent and adequate, and preparation for doing tasks (1). Prevalence and correlates of HRBs vary in different population groups. One of the ‘at-risk’ groups is people with Attention-Deficit/Hyperactivity Disorder (ADHD).

Almost hundred years ago, ADHD was described as a childhood disorder (especially in boys) called hyperactivity. Nearly six decades later, the "minimal brain damage" and "minimal brain dysfunction" were replaced with hyperactivity. With the introduction of attention deficit as the central feature of this disorder, a significant change occurred in the approaches of etiology and diagnosis of ADHD (2). Currently ADHD is diagnosed according to DSM-IV. Introducing diagnostic criteria and subtypes of ADHD (inattentive, hyperactive and combined subtypes) by APA (3), increased the convergence diagnosis and provided more appropriate frameworks for the studies on ADHD. The prevalence of ADHD is estimated at about 8-13% (4). Approximately 70% of children diagnosed with ADHD have the symptoms in adolescence (5), and a high percentage of these symptoms continue into adulthood (6). The prevalence of ADHD in adults is reported about 4.5% (7, 8).

People with ADHD, suffer from social and interpersonal problems. Inattention and hyperactive/impulsive behavior can cause social problems in this population (9), thus, People with ADHD suffer from more social and interpersonal problems. Compared with impulsive group, inattentive group is associated with less aggressive behaviors, conduct disorder and oppositional defiant behavior (10). Tobacco and drug usage (11), and smoking (12), are more common among children and adolescents with ADHD. The age of onset of smoking in people with ADHD is lower than those without a diagnosis of ADHD (13). In addition, there is a relationship between ADHD and high-risk driving (14). A study on 18-26 years old people with ADHD and without ADHD has shown that men with ADHD symptoms in childhood have experienced sexual activity and intercourse at a younger age and had more sexual partners (15), accident and adverse consequences of driving (14, 16).

Symptoms of ADHD, in general, and its comorbidity with other disorders (e.g. depression, phobias, substance abuse, and dysthymia) can increase probability of performing HRBs, but these are not the only determinants of high risk behaviors. Overall, ADHD patients have major problems in decision-making, executive functioning and social cognition. Their decision-making in ambiguous situations has the same features of "intuitive-existential" decision-making system (17). Also, the consequences of the decisions are not processed, and not applied in their decision- making. Additionally, people with ADHD are concerned with the anticipated reward of risk-seeking behavior and prefer these aspects (18). They also suffer from impairments in executive functions so their "rational-analytical system" is also disrupted. Some executive functions may be impaired in ADHD such as "response inhibition", "working memory", "set shifting" and" interference control" (18).

In a review of the studies done over a period of 30 years by Uekermann et al. found that ADHD is associated with deficit in social cognition (e.g., face perception, emotional prosody perception (19). Deficit in social cognition on one hand increases the probability of HRBs and on the other causes the reduced effects of treatment, particularly on depression and alcoholism, these disorders have high comorbidity with ADHD (20). 

Although our understanding of ADHD and its association with HRBs has increased by the conducted research, there are still questions and issues regarding to hereditary, neuropsychological, developmental, environmental, psychological, social factors of ADHD which should be studied further. Some of the major issues in this regard are listed below.

1- The interaction of genetic and environmental factors in the development of ADHD.

2- The development of brain networks and circuits associated with ADHD symptoms, impaired executive functions and social cognition of people with ADHD.

3- Deficits in executive function as a general capacity and also its components and how executive functions relate to HRBs in children and adults. 

4- Studying the effectiveness of drug treatments and psycho-social interventions on executive functions and improving the deficit of social cognition depending on ADHD subtypes and developmental stage of the patients.

5- Impact of parenting styles and cultural-social factors on development and maintenance of ADHD symptoms with HRBs, and the impact of ADHD on parental behaviors.

6- The role and influence of different situations and emotional states in decision-making of people with ADHD.
